# Editorial: Genetic horizons: exploring genetic biomarkers in therapy and evolution with the aid of artificial intelligence

**DOI:** 10.3389/fgene.2026.1775149

**Published:** 2026-02-06

**Authors:** Yiting Chen, Yiyin Zhang, Georgia Damoraki, Shu Wang

**Affiliations:** 1 Breast Center, Peking University People’s Hospital, Peking University, Beijing, China; 2 4th Department of Internal Medicine, Department of Medicine, School of Health Sciences, National and Kapodistrian University of Athens, Athens, Greece

**Keywords:** artificial intelligence (AI), deep learning, diagnostic models, drug prediction, genetic biomarkers, machine learning (ML), precision medicine

In the era of precision medicine, the intersection between AI and genetics holds transformative potential, could solve longstanding hurdles in genomic interpretation and biomarker-driven therapy. The sheer volume and multifaceted nature of multi-omics data often overwhelm conventional genetic frameworks, which remain ill-equipped to decode intricate disease networks, frequently stretching manual interpretation across grueling months. Current breakthroughs demonstrate AI’s capacity to reshape the field. Like, these studies include the Predictive Biomarker Modeling Framework (PBMF) has streamlined the discovery of actionable markers, effectively de-risking the high-stakes process of drug development (Arango-Argoty et al., 2025); An machine learning (ML) approach utilizes million-scale electronic health records to quantify pathogenic probabilities of over 1,600 genetic variants, replacing rigid binary classifications with continuous risk scores and overcoming biases from small-cohort analyses (Forrest et al., 2025). This Research Topic, “Genetic Horizons: Exploring Genetic Biomarkers in Therapy and Evolution with the aid of Artificial Intelligence”, aims to explore three core questions: how to leverage AI to overcome traditional genetic analysis limitations; how to address the gap of inefficient algorithms for seamless integration of laboratory and clinical data; and how to validate genomic algorithms via multicenter studies to enhance biomarker accuracy and biological relevance. This editorial summarizes key findings from featured articles, offering novel insights into genetic mechanisms through diverse experimental approaches.

Rather than struggling with the 'curse of dimensionality,’ ML leverages advanced architectures to parse vast mutation and expression arrays that are simply too intricate for traditional statistical frameworks to handle accurately. Pan et al. acute type A aortic dissection (ATAAD) study employs three synergistic ML algorithms (SVM-RFE, Random Forest, and LASSO) to screen six core genes from 676 differentially expressed genes, constructing a diagnostic model with an AUC of 0.94, far surpassing traditional single-gene biomarkers (AUC <0.8) (Pan et al.). Cui et al. osteoarthritis research adopts LASSO regression and SVM-RFE to identify 11 key methylated genes, achieving an AUC of 1.00 in the training set and 0.98 in the validation set, directly resolving high false-positive rates and ambiguous targets in traditional methylation analysis (Cui et al.). Xia et al. IgA nephropathy study utilizes an ensemble ML model integrating LASSO, Random Forest, and XGBoost to pinpoint biomarkers TYROBP and HCK, delivering a test set AUC of 0.942 by mitigating single-algorithm limitations (Xia et al.). These studies collectively demonstrate ML’s superiority in high-dimensional data processing for biomarker discovery.

All five studies prioritize genetic target exploration while relying on multi-dimensional data integration to enhance result reliability and clinical applicability, directly addressing the Research Topic’s second core question. Elasbali et al. Duchenne muscular dystrophy (DMD) study uses ML-derived computational tools to identify 50 deleterious mutations, 17 localizing to the CH1 domain (a critical actin-binding region), clarifying core pathogenic targets (Elasbali et al.). Foutadakis et al. enhancer review systematically summarizes ML’s role in deciphering disease-related regulatory circuits, categorizing tools like Enformer (for prediction) and DeepSTARR (for synthetic enhancers) to guide mechanistic research (Foutadakis et al.). Xia et al. study constructs a comprehensive “transcriptome-proteome-drug target” evidence chain, integrating self-built urinary RNA-seq data with 10 public datasets and validating via immunohistochemistry and molecular docking, representing a preliminary attempt at multi-omics and clinical data fusion (Xia et al.). Pan et al. ATAAD study validates core genes across three independent GEO datasets (e.g., GSE153434) and nine clinical samples, ensuring cross-cohort robustness (Pan et al.). Cui et al. osteoarthritis study complements this framework with methylation profiling to refine epigenetic targets (Cui et al.). While these efforts advance data integration, they still fall short of consistent multi-dimensional data fusion due to algorithmic inefficiencies.

Clinically oriented, these studies actively advance precision medicine by addressing unmet clinical needs. Pan et al. ATAAD nomogram, with 89.5% predictive accuracy, tackles the disease’s 21% 24-h mortality by offering a rapid emergency risk-assessment tool (Pan et al.). Xia et al. reported that urinary TYROBP and HCK serve as non-invasive biomarkers for IgA nephropathy and show a strong negative correlation with eGFR (R = −0.68), providing prognostic value (Xia et al.). Elasbali et al. DMD study identifies key mutations to guide gene-editing target selection, reducing mutation classification false-positives from 30% to 40% (traditional methods) to <10% (Elasbali et al.).

This editorial summarizes that AI-genetics integration, driven by ML algorithms, effectively overcomes traditional genetic analysis’ high-dimensional data processing bottlenecks. The five studies focus on disease-specific biomarker screening, multi-dimensional data integration (public datasets + clinical samples, multi-omics), and precision medicine advancement, exemplified by high-accuracy diagnostic models for ATAAD (AUC = 0.935) and IgA nephropathy (AUC = 0.942), and pathogenic mutation identification in DMD. Despite the significant strides documented here, several systemic hurdles must be cleared before these tools can be fully integrated into clinical practice. A primary obstacle remains the friction between disparate data types, although Xia et al. made a commendable preliminary attempt at multi-omics integration, the current lack of specialized algorithms continues to hinder the continuous, real-time synchronization required for clinical workflows (Xia et al.). Furthermore, the robustness of these findings is frequently constrained by a lack of large-scale, multicenter validation, as seen in the small cohorts of Cui et al. osteoarthritis study which may limit the generalizability of the results (Cui et al.). Beyond data volume, the “interpretability gap” remains a persistent concern: the “black-box” nature of many high-performing models means that even when core genes are identified as demonstrated in the ATAAD study by Pan et al., the specific mechanistic links to immune infiltration remain frustratingly opaque (Pan et al.). Finally, the applicability of these AI-driven genetic frameworks remains underexplored in rare diseases and diverse global populations, a gap that must be bridged to ensure equitable clinical translation. These gaps collectively hinder reliable clinical translation of AI-driven genetic research.

These studies collectively push forward AI’s application in genetic biomarker research, laying a foundation for precision medicine’s clinical translation. We invite readers from diverse perspectives to continue the conversation and share feedback to address remaining challenges.
